# The relationship between self-disorders (SDS) and depressive/anxious symptoms in a clinical sample of adolescents

**DOI:** 10.1192/j.eurpsy.2021.1225

**Published:** 2021-08-13

**Authors:** E. Monducci, G. Colafrancesco, C. Battaglia, E. Masini, G. De Vita, M. Ferrara

**Affiliations:** 1 Department Of Human Neurosciences, University of Rome Sapienza, Rome, Italy; 2 Department Of Human Neurosciences,section Of Child And Adolescent Neuropsychiatry, University of Rome Sapienza, Rome, Italy

**Keywords:** Anxiety, Depression, Self-Disorders, Schizotypal personality disorder

## Abstract

**Introduction:**

Self-disorders (SDs) have been described as a core feature of schizophrenia-spectrum disorders. Previous studies conducted on heterogeneous clinical adult and adolescents samples demonstrated that SDs aggregate selectively in the schizophrenia spectrum disorders compared to other disorders.

**Objectives:**

To examine the specificity of SDs for schizophrenia spectrum disorders in adolescent inpatient sample.

**Methods:**

Fifty-five adolescent inpatients admitted to the Child Psychiatry Unit at the Sapienza University in Rome were assessed for psychopathology using Kiddie Schedule for Affective Disorders and Schizophrenia (K-SADS-PL), Structured Interview for Prodromal Syndromes (SIPS/SOPS),Examination of Anomalous Self-Experiences (EASE), Multidimensional Anxiety Scale for Children (MASC), Calgary depression scale for schizophrenia (CDSS)

**Results:**

Patients, aged 14-18 years, were divided in four diagnostic groups: schizophrenia spectrum disorders (5 pts.), mood disorders (19 pts.), anxiety disorders (27 pts.) and other disorders (4 pts.). Frequency of self-disorders was different among the 4 groups. Including patients schizotypal personality disorder in the schizophrenia-spectrum disorder group, the difference is still significant. Mann-Whitney U test shows no differences between EOP and UHR patients in SD. Furthermore, correlations between EASE total score and Calgary and MASC total scores were significant.

**Conclusions:**

Our results confirm the specificity of SDs for schizophrenia spectrum disorders and also the belonging of schizotypal personality disorder to schizophrenia-spectrum.

